# Supplementary material for: Meningococcal Deduced Vaccine Antigen Reactivity (MenDeVAR) Index: a Rapid and Accessible Tool That Exploits Genomic Data in Public Health and Clinical Microbiology Applications
Source: J Clin Microbiol. 2020 Dec 17;59(1):e02161-20. doi: 10.1128/JCM.02161-20 (PMC7771438; doi:10.1128/JCM.02161-20)
Supplement: Supplemental file 1 [file JCM.02161-20-s0001.pdf]

Supplemental data

Table S1: Experimental studies identified through literature search to determine the cross-reactive antigenic variants to Bexsero® (4CMenB) vaccine included for combined analysis.

Study	Year	Assay	Country of isolate collection	No. of meningococcal isolates studied	Reference
Abad *et al*.	2016	MATS	Spain	300	1
Bettinger *et al*.	2013	MATS	Canada	157	2
Budroni *et al*.	2016	MATS	UK	40	3
Mowlabococcus *et al*.	2016	MATS	Australia	278	4
Mulhall *et al*.	2018	MATS	Republic of Ireland	105	5
Parikh *et al*.	2017	MATS	UK	251	6
Rajam *et al*.	2017	MATS	US	442	7
Vogel *et al*.	2013	MATS	European	1052	8
Wasko *et al*.	2016	MATS	Poland	196	9
Abad *et al*.	2015	MATS	Spain	10	10
Green *et al*.	2018	MATS	UK	69	11
Kesanopoulos *et al*.	2018	MATS	Greece	71	12
Stella *et al*.	2019	MATS	UK	34	13

Table S2: Experimental studies identified through literature search to determine the cross-reactive antigenic variants to Trumenba® (rLP2086) vaccine included for combined analysis.

**Study**	**Year**	**Assay**	**Demographic of study participants**	**No. of study participants**	**Reference**
McNeil *et al.*	2018	MEASURE	Adults	Not relevant	14
Harris *et al.*	2017	SBA	Adolescents and adults	Not specified	15
Lujan *et al.*	2017	SBA	Healthcare workers	17	16
Taha *et al.*	2017	SBA	11-18 year olds	15	17
Ostergaard *et al.*	2017	SBA	Adolescent, young adults	3596, 3304	18
Marshall *et al.*	2016	SBA	11-18 year olds	539	19
Muse *et al.*	2016	SBA	10-13 year old children	2648	20
Senders *et al.*	2016	SBA	11-17 year old children	2499	21
Vesikari *et al.*	2016	SBA	11-18 year old children	749	22
Reiner *et al.*	2016	SBA	Healthcare workers	7	23
Marshall *et al.*	2013	SBA	Adults	60	24
Vesikari *et al.*	2016	SBA	11-18 year olds	1714	25
Nissen *et al.*	2016	SBA	8-14 year old children	127	26
Richmond *et al.*	2012	SBA	18-25 year old adults	130	27
Richmond *et al.*	2012	SBA	Adolescents	538	28
Marshall *et al.*	2012	SBA	Healthy toddlers	99	29

References

1. Abad R, Medina V, Stella M, Boccadifuoco G, Comanducci M, Bambini S, Muzzi A, Vazquez JA. 2016. Predicted Strain Coverage of a New Meningococcal Multicomponent Vaccine (4CMenB) in Spain: Analysis of the Differences with Other European Countries. PLoS One 11:e0150721.
2. Bettinger JA, Scheifele DW, Le Saux N, Halperin SA, Vaudry W, Tsang R. 2013. The disease burden of invasive meningococcal serogroup B disease in Canada. Pediatric Infectious Disease Journal 32:e20-e25.
3. Budroni S, Kleinschmidt A, Boucher P, Medini D. 2016. Pooled-sera hSBA titres predict individual seroprotection in infants and toddlers vaccinated with 4CMenB. Vaccine 34:2579-84.
4. Mowlaboccus S, Perkins TT, Smith H, Sloots T, Tozer S, Prempeh LJ, Tay CY, Peters F, Speers D, Keil AD, Kahler CM. 2016. Temporal Changes in BEXSERO® Antigen Sequence Type Associated with Genetic Lineages of *Neisseria meningitidis* over a 15-Year Period in Western Australia. PLoS One 11:e0158315.
5. Mulhall RM, Bennett D, Cunney R, Borrow R, Lucidarme J, Findlow J, Jolley KA, Bray J, Maiden MCJ, Moschioni M, Serino L, Stella M, Medini D. 2018. Potential Coverage of the 4CMenB Vaccine against Invasive Serogroup B *Neisseria meningitidis* Isolated from 2009 to 2013 in the Republic of Ireland. mSphere 3.
6. Parikh SR, Newbold L, Slater S, Stella M, Moschioni M, Lucidarme J, De Paola R, Giuliani M, Serino L, Gray SJ, Clark SA, Findlow J, Pizza M, Ramsay ME, Ladhani SN, Borrow R. 2017. Meningococcal serogroup B strain coverage of the multicomponent 4CMenB vaccine with corresponding regional distribution and clinical characteristics in England, Wales, and Northern Ireland, 2007-08 and 2014-15: a qualitative and quantitative assessment. Lancet Infectious Diseases 17:754-762.
7. Rajam G, Stella M, Kim E, Paulos S, Boccadifuoco G, Serino L, Carlone G, Medini D. 2017. Meningococcal Antigen Typing System (MATS)-Based Neisseria meningitidis Serogroup B Coverage Prediction for the MenB-4C Vaccine in the United States. mSphere 2.
8. Vogel U, Taha MK, Vazquez JA, Findlow J, Claus H, Stefanelli P, Caugant DA, Kriz P, Abad R, Bambini S, Carannante A, Deghmane AE, Fazio C, Frosch M, Frosi G, Gilchrist S, Giuliani MM, Hong E, Ledroit M, Lovaglio PG, Lucidarme J, Musilek M, Muzzi A, Oksnes J, Rigat F, Orlandi L, Stella M, Thompson D, Pizza M, Rappuoli R, Serruto D, Comanducci M, Boccadifuoco G, Donnelly JJ, Medini D, Borrow R. 2013. Predicted strain coverage of a meningococcal multicomponent vaccine (4CMenB) in Europe: a qualitative and quantitative assessment. Lancet Infectious Diseases 13:416-425.
9. Wasko I, Hong E, De Paola R, Stella M, Moschioni M, Taha MK, Skoczynska A. 2016. High predicted strain coverage by the multicomponent meningococcal serogroup B vaccine (4CMenB) in Poland. Vaccine 34:510-515.
10. Abad R, Biolchi A, Moschioni M, Giuliani MM, Pizza M, Vazquez JA. 2015. A large portion of meningococcal antigen typing system-negative meningococcal strains from spain is killed by sera from adolescents and infants immunized with 4CMenB. Clinical and Vaccine Immunology 22:357-60.
11. Green LR, Lucidarme J, Dave N, Chan H, Clark S, Borrow R, Bayliss CD. 2018. Phase Variation of NadA in Invasive Neisseria meningitidis Isolates Impacts on Coverage Estimates for 4C-MenB, a MenB Vaccine. Journal of Clinical Microbiology 56.
12. Kesanopoulos K, Bratcher HB, Hong E, Xirogianni A, Papandreou A, Taha MK, Maiden MCJ, Tzanakaki G. 2018. Characterization of meningococcal carriage isolates from Greece by whole genome sequencing: Implications for 4CMenB vaccine implementation. PLoS One 13:e0209919.
13. Stella M, Giuliani M, Biolchi A, Tomei S, De Paola R, Bai X, Borrow R, Lucidarme J, La Gaetana R, Toneatto D, Pizza M, Serino L, Mori E, Giuliani MM. 2020. Does vaccination with 4CMenB convey protection against meningococcal serogroup B strains not predicted to be covered by MATS? A study of the UK clonal complex cc269. Human Vaccines & Immunotherapy 16:945-948.

14. McNeil LK, Donald RGK, Gribenko A, French R, Lambert N, Harris SL, Jones TR, Li S, Zlotnick G, Vogel U, Claus H, Abad R, Vazquez JA, Borrow R, Findlow J, Taha MK, Deghmane AE, Caugant DA, Kriz P, Musilek M, Wang X, Vuong J, Mayer LW, Pride MW, Jansen KU, Anderson AS. 2018. Predicting the Susceptibility of Meningococcal Serogroup B Isolates to Bactericidal Antibodies Elicited by Bivalent rLP2086, a Novel Prophylactic Vaccine. MBio 9.
15. Harris SL, Donald RG, Hawkins JC, Tan C, O'Neill R, McNeil LK, Perez JL, Anderson AS, Jansen KU, Jones TR. 2017. Neisseria meningitidis Serogroup B Vaccine, Bivalent rLP2086, Induces Broad Serum Bactericidal Activity Against Diverse Invasive Disease Strains Including Outbreak Strains. Pediatric Infectious Diseases Journal 36:216-223.
16. Lujan E, Partridge E, Giuntini S, Ram S, Granoff DM. 2017. Breadth and Duration of Meningococcal Serum Bactericidal Activity in Health Care Workers and Microbiologists Immunized with the MenB-FHbp Vaccine. Clinical and Vaccine Immunology 24.
17. Taha MK, Hawkins JC, Liberator P, Deghmane AE, Andrew L, Hao L, Jones TR, McNeil LK, O'Neill RE, Perez JL, Jansen KU, Anderson AS. 2017. Bactericidal activity of sera from adolescents vaccinated with bivalent rLP2086 against meningococcal serogroup B outbreak strains from France. Vaccine 35:1530-1537.
18. Ostergaard L, Vesikari T, Absalon J, Beeslaar J, Ward BJ, Senders S, Eiden JJ, Jansen KU, Anderson AS, York LJ, Jones TR, Harris SL, O'Neill R, Radley D, Maansson R, Pregaldien JL, Ginis J, Staerke NB, Perez JL, B, Investigators BT. 2017. A Bivalent Meningococcal B Vaccine in Adolescents and Young Adults. New England Journal of Medicine 377:2349-2362.
19. Marshall HS, Richmond PC, Beeslaar J, Jiang Q, Jansen KU, Garces-Sanchez M, Martinon-Torres F, Szenborn L, Wysocki J, Eiden J, Harris SL, Jones TR, Lee SS, Perez JL, Investigators AS. 2017. Meningococcal serogroup B-specific responses after vaccination with bivalent rLP2086: 4 year follow-up of a randomised, single-blind, placebo-controlled, phase 2 trial. Lancet Infect Dis 17:58-67.
20. Muse D, Christensen S, Bhuyan P, Absalon J, Eiden JJ, Jones TR, York LJ, Jansen KU, O'Neill RE, Harris SL, Perez JL. 2016. A Phase 2, Randomized, Active-controlled, Observer-blinded Study to Assess the Immunogenicity, Tolerability and Safety of Bivalent rLP2086, a Meningococcal Serogroup B Vaccine, Coadministered With Tetanus, Diphtheria and Acellular Pertussis Vaccine and Serogroup A, C, Y and W-135 Meningococcal Conjugate Vaccine in Healthy US Adolescents. Pediatric Infectious Diseases Journal 35:673-82.
21. Senders S, Bhuyan P, Jiang Q, Absalon J, Eiden JJ, Jones TR, York LJ, Jansen KU, O'Neill RE, Harris SL, Ginis J, Perez JL. 2016. Immunogenicity, Tolerability and Safety in Adolescents of Bivalent rLP2086, a Meningococcal Serogroup B Vaccine, Coadministered with Quadrivalent Human Papilloma Virus Vaccine. Pediatric Infectious Diseases Journal 35:548-54.
22. Vesikari T, Wysocki J, Beeslaar J, Eiden J, Jiang Q, Jansen KU, Jones TR, Harris SL, O'Neill RE, York LJ, Perez JL. 2016. Immunogenicity, Safety, and Tolerability of Bivalent rLP2086 Meningococcal Group B Vaccine Administered Concomitantly With Diphtheria, Tetanus, and Acellular Pertussis and Inactivated Poliomyelitis Vaccines to Healthy Adolescents. Journal of the Pediatric Infectious Disease Society 5:180-7.
23. Reiner DM, Bhuyan P, Eiden JJ, Ginis J, Harris S, Jansen KU, Jiang Q, Jones TR, O'Neill RE, York LJ, Perez JL. 2016. Immunogenicity, safety, and tolerability of the meningococcal serogroup B bivalent rLP2086 vaccine in adult laboratory workers. Vaccine 34:809-13.
24. Marshall HS, Richmond PC, Nissen MD, Wouters A, Baber J, Jiang Q, Anderson AS, Jones TR, Harris SL, Jansen KU, Perez JL. 2013. A phase 2 open-label safety and immunogenicity study of a meningococcal B bivalent rLP2086 vaccine in healthy adults. Vaccine 31:1569-75.
25. Vesikari T, Ostergaard L, Diez-Domingo J, Wysocki J, Flodmark CE, Beeslaar J, Eiden J, Jiang Q, Jansen KU, Jones TR, Harris SL, O'Neill RE, York LJ, Crowther G, Perez JL. 2016. Meningococcal Serogroup B Bivalent rLP2086 Vaccine Elicits Broad and Robust Serum Bactericidal Responses in Healthy Adolescents. Journal of the Pediatric Infectious Disease Society 5:152-60.

26. Nissen MD, Marshall HS, Richmond PC, Jiang Q, Harris SL, Jones TR, Jansen KU, Perez JL. 2013. A randomized, controlled, phase 1/2 trial of a Neisseria meningitidis serogroup B bivalent rLP2086 vaccine in healthy children and adolescents. Pediatr Infect Dis J 32:364-71.
27. Richmond PC, Nissen MD, Marshall HS, Lambert SB, Roberton D, Gruber WC, Jones TR, Arora A. 2012. A bivalent Neisseria meningitidis recombinant lipidated factor H binding protein vaccine in young adults: results of a randomised, controlled, dose-escalation phase 1 trial. Vaccine 30:6163-74.
28. Richmond PC, Marshall HS, Nissen MD, Jiang Q, Jansen KU, Garces-Sanchez M, Martinon-Torres F, Beeslaar J, Szenborn L, Wysocki J, Eiden J, Harris SL, Jones TR, Perez JL. 2012. Safety, immunogenicity, and tolerability of meningococcal serogroup B bivalent recombinant lipoprotein 2086 vaccine in healthy adolescents: a randomised, single-blind, placebo-controlled, phase 2 trial. Lancet Infectious Diseases 12:597-607.
29. Marshall HS, Richmond PC, Nissen MD, Jiang Q, Anderson AS, Jansen KU, Reynolds G, Ziegler JB, Harris SL, Jones TR, Perez JL. 2012. Safety and Immunogenicity of a Meningococcal B Bivalent rLP2086 Vaccine in Healthy Toddlers Aged 18-36 Months: A Phase 1 Randomized-controlled Clinical Trial. Pediatr Infect Dis J 31:1061-1068.
